# Evidence for methane in Martian meteorites

**DOI:** 10.1038/ncomms8399

**Published:** 2015-06-16

**Authors:** Nigel J. F. Blamey, John Parnell, Sean McMahon, Darren F. Mark, Tim Tomkinson, Martin Lee, Jared Shivak, Matthew R. M. Izawa, Neil R. Banerjee, Roberta L. Flemming

**Affiliations:** 1 Department of Earth Sciences, Brock University, 500 Glenridge Avenue, St Catharines, Ontario, Canada L2S 3A1; 2 School of Geosciences, University of Aberdeen, Aberdeen AB24 3UE, UK; 3 Scottish Universities Environmental Research Centre, Glasgow G75 0QF, UK; 4 School of Geographical and Earth Sciences, University of Glasgow, Glasgow G12 8QQ, UK; 5 Department of Earth Sciences, University of Western Ontario, Ontario, Canada N6A 5B7

## Abstract

The putative occurrence of methane in the Martian atmosphere has had a major influence on the exploration of Mars, especially by the implication of active biology. The occurrence has not been borne out by measurements of atmosphere by the MSL rover Curiosity but, as on Earth, methane on Mars is most likely in the subsurface of the crust. Serpentinization of olivine-bearing rocks, to yield hydrogen that may further react with carbon-bearing species, has been widely invoked as a source of methane on Mars, but this possibility has not hitherto been tested. Here we show that some Martian meteorites, representing basic igneous rocks, liberate a methane-rich volatile component on crushing. The occurrence of methane in Martian rock samples adds strong weight to models whereby any life on Mars is/was likely to be resident in a subsurface habitat, where methane could be a source of energy and carbon for microbial activity.

## Abstract (web summary)

Extremophiles on Earth are known to respire methane, and the potential existence of methane on Mars indicates similar organisms could survive there. Here, the authors present data from Martian meteorites confirming the presence of methane, indicating that a habitat capable of supporting organisms exists on Mars.

## Graphical abstract

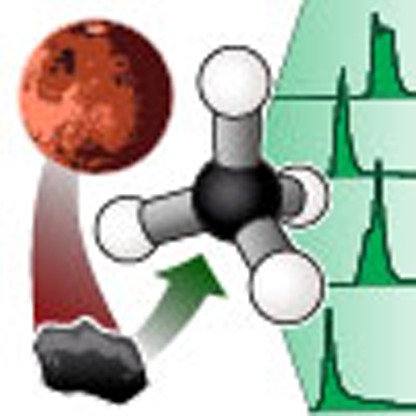

On Earth, methane (CH_4_) can have a microbial origin (methanogenesis) and/or is a source of carbon and metabolic energy for microbes (methanotrophs); hence, the putative occurrence of methane in the Martian atmosphere^1^,^2^ attracted much attention for its possible biological significance^3^,^4^,^5^,^6^. However, alternative treatments of the data have raised uncertainty about the occurrence of methane^7^,^8^. Nevertheless, analysis of the local Martian atmosphere by the Mars Science Laboratory (MSL) rover Curiosity has detected transient methane anomalies^9^. Any life on Mars is likely to be in the subsurface^10^,^11^,^12^, and the potential remains for a subsurface habitat based on methane derived inorganically from low-temperature (<100 °C) reactions of water or carbon-bearing fluids with basalt and other rocks^13^. The serpentinization of olivine and hydration of pyroxene in basalt and other rocks in the presence of water yields hydrogen, which in turn may interact with carbon-bearing species such as carbon monoxide (CO), carbon dioxide (CO_2_) and formic acid (HCOOH) to yield methane^3^,^14^,^15^,^16^.

Methane in submarine terrestrial basalts can support a microbial population^17^. There are also large volumes of basalt and other basic igneous rocks on Mars^18^. Olivine-rich volcanic rocks have been identified in each of the Noachian, Hesperian and Amazonian successions, and olivine is a significant component of Martian sediment, unlike on Earth^19^. Pyroxene is volumetrically greater than olivine within the Martian crust, and is potentially important as, in addition to a source of hydrogen^20^, on Earth altered pyroxenes harbour life^21^. Widespread hydrated silicates on Mars imply extensive water–rock interaction^22^, and the opportunities for gas-generating alteration in the Martian crust should, therefore, be widespread. The olivine in Martian meteorites, including all of the nakhlites and many of the shergottites, has experienced aqueous alteration including serpentinization^23^. Serpentinization of olivine at some stage in the past is also recorded on Mars through orbital measurements, using MRO-CRISM^24^,^25^ and could be extensive^10^. Martian meteorites contain magmatic carbon, carbonate carbon from interaction with Martian atmospheric CO_2_ and organic carbon from meteoritic infall^26^. We anticipate that all of these carbon-bearing components could become entrained in hydration reactions, which alter olivine and pyroxene, and contribute to methane generation.

There is an opportunity to assess whether Martian rocks could be releasing methane by the analysis of Martian meteorites. The volatile components of the meteorites must be derived from a mixture of sources, including Martian atmosphere, Martian magmatism, Martian crustal processes and terrestrial contamination^27^. All meteorites from Mars have a bulk basic or ultrabasic igneous composition^18^; hence, it is possible that some of them have generated methane upon aqueous alteration before ejection from Mars. A range of other fluid-related signatures obtained from Martian meteorites (for example, refs 28, 29, 30, 31) implies that methane generated from alteration on Mars could survive the journey to Earth and be amenable to extraction.

The analysis of entrapped gas has been undertaken on six Martian meteorites (Supplementary Table 1, Supplementary Note 1). These were nakhlites Nakhla, Y 000749, NWA 5790 and MIL 03346, and the shergottites Zagami and LA002. The nakhlites are known to have experienced extensive alteration in a highly oxidizing environment on Mars, and many shergottites show evidence of some interaction with oxidizing fluids in the subsurface^28^. Nakhla and the shergottites do not exhibit significant terrestrial weathering^28^,^32^; however, MIL 03346, NWA 5790 and Y 000749 show evidence of significant aqueous alteration on both Mars and Earth^31^,^33^,^34^,^35^. With the exception of NWA 5790, the samples were from meteorite interiors, to mitigate against terrestrial contamination. All samples excluded any fusion crust, so they should not have been thermally altered during atmospheric entry (Supplementary Note 2), and fluid inclusions should remain intact^36^. The meteorites were analysed using the crush-fast scan technique, in which incremental crushing at room temperature liberates gases trapped in fluid inclusions and crystal boundaries, into a quadrupole mass spectrometer^37^. Each crush yields a burst of gas that is individually analysed. Gas compositions are calibrated against standard gas mixtures and fluid inclusion standards of known composition. This technique has the advantage that it does not involve the liberation of gas through heating, in which carbon-bearing components could be converted into a different form. Thus, carbonates, which would yield a strongly CO_2_-rich signature due to thermal breakdown upon heating, can yield a CH_4_-rich signature if that is the predominant entrapped gas. Analyses of gases in meteorites following the heating of samples provide valuable information, including the isotopic composition of the carbon reservoir^38^, and liberation of entrained hydrocarbons^28^, but preclude detailed determination of the indigenous carbon-bearing gas species.

## Results

### Gases in Martian meteorites

**Figure 1:**
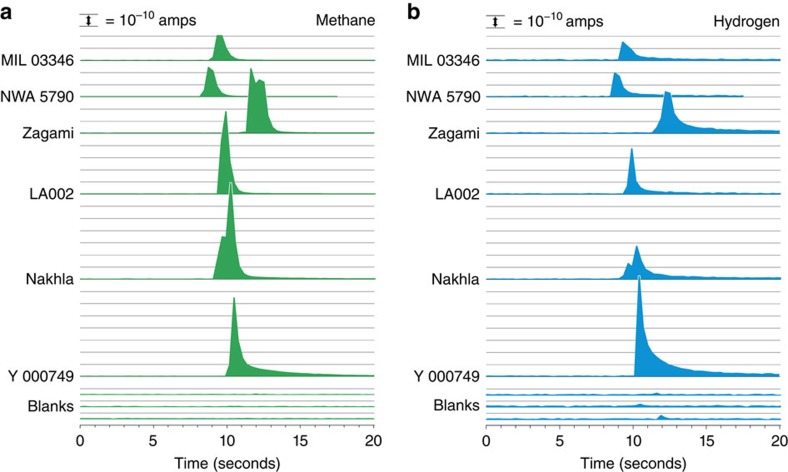
Output from crush fast scan-mass spectrometry analyses of Martian meteorites. Output shown from during CH_4_ (**a**) and H_2_ (**b**) release. Output is over 80 cycles (∼20–25 s) at *m/z*=15 and *m/z*=2, representing ionized CH_3_+ and H_2_+ during sample crushing. The ionized volatiles produce an instantaneous current in the detector proportional to their amount. The signals from Martian meteorites are shown above quartz blanks, in units of 10^−10^ amps above background. Hydrogen generates more current than an equal volume of methane because of a difference in sensitivity factor. Doublet peaks may represent gas release from successively crushed inclusions. Peaks are displaced laterally for clarity only. The meteorite methane peak signals are 2 to 3 orders of magnitude higher than the highest signal from the blank.

**Figure 2:**
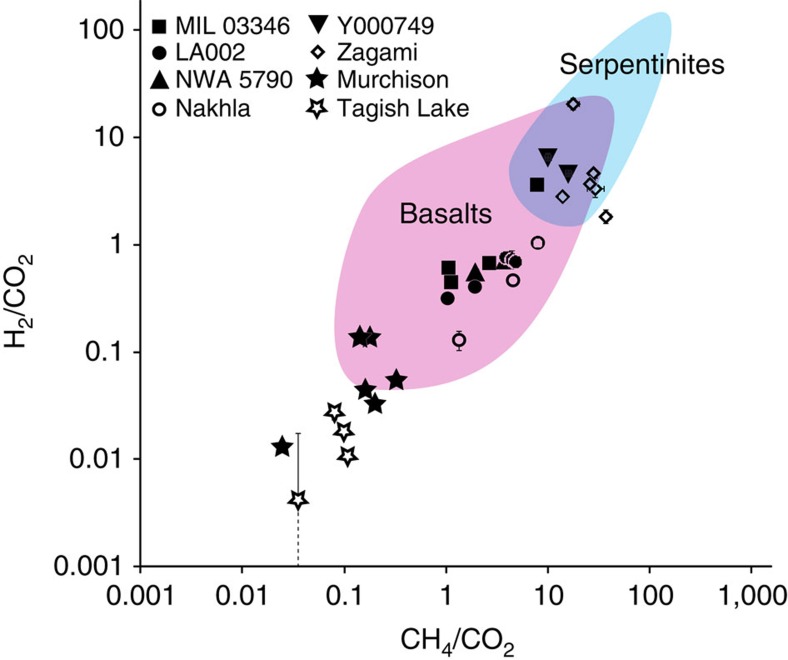
Comparison of gas compositions in Martian meteorites with those in terrestrial samples and carbonaceous chondrites. The terrestrial samples are from basalts and terrestrial fluids. Each point corresponds to one burst of volatiles produced by an increment of crushing. Gases of interest are normalized to CO_2_. All Martian meteorites plot within the data field for terrestrial basalts; both are more methane-rich than terrestrial fluids and carbonaceous chondrites. Errors are obtained from molar volumes of individual species±one weighted s.d. (see Methods). One Tagish Lake analysis has large error bar, but in most cases error bars are smaller than data symbols. Data sources: terrestrial serpentinites^39^, and terrestrial basalts^45^, both crushed in same manner as the meteorites.

**Figure 3:**
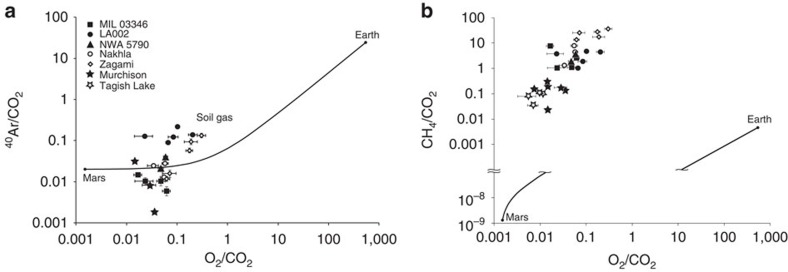
Comparison of Martian meteorite data with mixing lines between Martian and terrestrial atmospheres. Data are from ref. 27. Each point corresponds to one burst of volatiles produced by an increment of crushing. Gases of interest are normalized to CO_2_. Ar/O_2_ plot (**a**) implies a primary Martian signature with limited contamination from the terrestrial atmosphere. CH_4_/O_2_ plot (**b**) implies substantial enrichment in CH_4_ by a non-atmospheric source, most likely in the Martian crust. The Ar/O_2_ meteorite data lie close to the composition of terrestrial soil gas, but the CH_4_ enrichment excludes soil gas as a major contaminant. Together the plots show that contamination from terrestrial atmosphere was minimal, and that the gas signature is crustal rather than atmospheric. Errors are obtained from molar volumes of individual species±one weighted s.d. (see Methods).

The gases released were dominated by CH_4_, CO_2_, H_2_, N_2_, with traces of O_2_ and Ar. The highest yields were from Nakhla and LA 002 and lowest from NWA 5790 and MIL 03346 (Fig. 1; Supplementary Table 2). The CH_4_/CO_2_ ratio varies from about 3 (LA002, NWA 5790, MIL 03346) to 23 (Zagami). The H_2_/CO_2_ ratio varies from 0.5 to 7. These values are all in the same range as those for terrestrial basalts (Fig. 2). The lower CH_4_/CO_2_ ratio for Martian samples compared with terrestrial serpentinites^39^ may reflect an abundance of CO_2_ in the Martian crust, evidenced by carbonation observed in meteorites^40^ and from orbit^41^. Variations in H_2_/CH_4_ ratio imply varying degrees of interaction between H_2_ and available carbon, but would also reflect differential retention of H_2_ and CH_4_ in the rock. The artificial generation of CH_4_ during application of the steel crushing device was monitored using procedural blanks of heat-degassed basalt, as previous studies showed that crushing creates traces of CH_4_ (refs 42, 43, 44). The contributions of the blanks to the methane found in the meteorite samples (Fig. 1) were 0.03 to 0.1% (mean 0.07%) for the mean blank, and 0.7 to 2.3% for the maximum blank of mean value plus s.d. The contribution of the blank to the methane measured in the Nakhla sample is 0.05%. Normalization of gases to CO_2_ and comparison with the composition of the terrestrial and Martian atmosphere^27^ allows the plotting of data relative to mixing lines between the two atmospheres (Fig. 3). A plot of argon against oxygen shows that the meteorite data plots much closer to the Martian atmosphere composition with Ar/O_2_ ratio ∼13. Contamination by the terrestrial atmosphere, which has Ar/O_2_ ratio ∼0.05, would shift the data along the mixing line away from the Martian end member. The proximity of the meteorite data to the Martian end member indicates that terrestrial contamination is limited, although the composition of terrestrial soil gas lies closer to the meteorite data than the composition of the terrestrial atmosphere. However, a plot of methane against oxygen shows meteorite data well removed from the atmospheric mixing line (Fig. 3). There is substantially more methane than in the Martian atmosphere, strongly suggesting a mechanism for methane generation in the Martian crust. An alternative explanation could be that the methane composition of the atmosphere was much higher when the nakhlites were being altered about 600 Myr ago, but the similar enrichment in terrestrial basalts suggests that a crustal origin is more likely. The scatter in the data reflects variations in mineralogy including proportions of alteration products, and gas retention, and is comparable with the scatter found in terrestrial basalts^45^, as shown in Fig. 2.

## Discussion

The gases released by crushing are likely to be resident within fluid inclusions, which have been observed in nakhlite meteorites^29^, and crystal boundaries. The relatively high hydrogen and methane contents are consistent with the aqueous alteration of olivine and pyroxene in the meteorites. Nakhlites in particular are known to have experienced significant aqueous alteration on Mars, including the formation of carbonates, sulfates and hydrous silicates^23^. Olivines found within the nakhlites and other Martian meteorites are iron-rich compared with typical terrestrial olivines, and are therefore particularly susceptible to serpentinization reactions^3^,^14^. Indeed, traces of serpentine have been detected in the Yamato 000593 meteorite^23^. Therefore, hydrogen and methane should have been generated from these rocks on Mars, just as they are in comparable rocks on Earth. There is a strong implication that the gases measured in this study are predominantly Martian in origin.

Several other lines of evidence suggest that the gases had a Martian origin.

First, the samples that had experienced most alteration, both on Mars and Earth, have the lowest gas yields. This suggests that alteration had facilitated gas release, either during the alteration process or during subsequent history. This does not preclude the adsorption of gas after arrival on Earth, but the much greater gas yields from the less altered meteorites, including the shergottites, implies that their gas signatures are largely Martian in origin.

Second, the gas assemblages in the meteorites compare closely with those from terrestrial basalts, that is, the meteorites contain those gases expected from basaltic rocks (Fig. 2), suggesting a similar genesis by alteration of the basalt mineralogy. Measurements on terrestrial basalts and serpentinites show that they consistently yield gas with high CH_4_/CO_2_ ratios, commonly >1 (refs 39, 45). Many other data sets measured by the same technique yield very different assemblages^37^. These data sets underline the CH_4_-rich signature (CH_4_/CO_2_>5) for the meteorite basalts. If the gas assemblage was modified on Mars, for example by uptake of atmospheric CO_2_ in secondary carbonates, or during the shock of meteorite ejection^26^, the CH_4_/CO_2_ ratio would have been lowered from the original composition, further emphasizing the significance of the CH_4_ as a primary signature of basalt alteration.

Third, samples of a group of meteorites with a very different composition, carbonaceous chondrites (Murchison, Tagish Lake), yield a distinct gas assemblage, characterized by 1 to 2 orders of magnitude lower CH_4_/CO_2_ and H_2_/CO_2_ (Fig. 2; data from this study, in Supplementary Table 2). Both groups of meteorite have experienced low-temperature alteration of silicates, probably at low water–rock ratios^46^. The carbonaceous chondrites differ in containing organic carbon at the percentage level, orders of magnitude greater than in the Martian meteorites. This suggests that the gas assemblages are a product of the meteorite mineralogy, rather than a characteristic of the terrestrial environment in which the meteorites arrived.

There is evidence for the adsorption of terrestrial air^47^ and for a terrestrial component of carbon^26^,^28^ in Martian meteorites. The incorporation of air in surface environments on Earth can result in measurable levels of oxygen in the gases liberated on crushing, including in samples of oxidized basalt^45^. Traces of oxygen occur in the meteorites, most abundantly in those meteorites known to have experienced terrestrial weathering, MIL 03346 and NWA 5790. This observation indicates that some interaction with terrestrial air may have occurred, but terrestrial air or soil gas would not contribute the methane-rich gas assemblage. It might be argued that some interaction with water could have occurred since arrival on Earth. However, in the gas-rich Nakhla and Zagami samples, it is unrealistic to envisage this gas generation on the timescales since they fell to Earth (1911, 1962). Traces of oxygen also occur in the Martian atmosphere, but the meteorites do not exhibit the high Ar content in the Martian atmosphere. We can estimate the possible degree of contamination, based on the oxygen contents of the meteorite gases. One meteorite (Y000749) has no oxygen, and effectively represents a Martian basalt end member, while the terrestrial atmosphere has ∼21%. The percentage oxygen in the other meteorites (ranging up to 0.72%) would be the equivalent of up to 3.4% contamination by the terrestrial atmosphere.

The availability of methane and hydrogen is critical to the potential of the Martian crust as a habitat for microbial life. The hostile Martian surface is probably less habitable than the subsurface, and several scenarios have been proposed for deep Martian life^10^,^11^,^12^. The gases would be most concentrated in subsurface environments such as fracture systems and basalt lava vesicles, where they could support a deep biosphere, as envisaged in basalts and other crystalline basement rocks on Earth^14^,^48^. Methane supports the deep biosphere on Earth, including in basalt where, critically for a Martian analogue, the methane is used by anaerobic microbes and may be partially coupled to the microbial cycling of sulfur^17^. The evidence presented here indicates that a methane-bearing subsurface habitat is similarly available on Mars. Whether or not the habitat has been occupied remains to be determined.

## Methods

### Sample processing

Extraction and analysis of volatiles was performed by the crush-fast scan (CFS) method^37^,^49^, which offers very low detection limits^50^. Samples the size of a match head (that is, milligram to gram scale) were first dried below 100 °C. Samples were then loaded into a crushing chamber then held under vacuum (∼10^−8^ Torr) for 72 h. The samples were crushed in swift increments, with each crush producing a burst of mixed volatiles which is individually analysed. A typical sample size of about 250 mg (one or two 3-mm grains) released 4–10 bursts of volatiles (up to ∼2 × 10^−11^ mol) into the vacuum chamber, which remained there for 8–10 analyser scans (∼2 s) before removal by the vacuum pump. This method does not require a carrier gas and volatiles are not separated from each other but released simultaneously into the chamber. Each individual crush was analysed for H_2_, He, CH_4_, N_2_, O_2_, Ar and CO_2_, using a Pfeiffer Prisma ‘residual-gas' quadrupole mass spectrometer operating in fast-scan, peak-hopping mode at room temperature. In multiple ion detection mode, both isotopic peaks and fragmentation peaks are observed; the major peaks for individual gases are generally selected but some exceptions occur. Methane is detected at mass 15 (that is, the CH_3_+ fragment) to avoid interference by O- fragments from water (the methane peak at mass 15 is about 90% as intense as the peak at mass 16). CH_3_+ fragments generated by other organic compounds are typically much less abundant and can be corrected for. It is likely that the meteorites host multiple fluid inclusions and, as the sample is incrementally crushed, inclusions can rupture several milliseconds apart to produce a doublet in some mass scans, as seen in Fig. 1. Ratios of gases are reported, as the crushing does not liberate the total amount of entrapped gas. The instrument was calibrated using Scott Gas Mini-mix commercial standard gas mixtures, synthetic inclusions filled with gas mixtures and three in-house fluid inclusion gas standards^49^. The Mini-mix gas mixtures have a possible 2% error in the value of the secondary gas mixed with hydrogen. Data are reported on a water-free basis. Instrumental blanks are also analysed routinely. The amount of each species was calculated by matrix methods^51^,^52^ to provide a quantitative analysis, which is corrected for the instrumental background. Crushing does not liberate all the entrapped gas from samples, so data are generated as molar percentages rather than moles. To circumvent the dependence of individual percentages on irrelevant species, we report methane abundances as the ratio (mol% CH_4_)/(mol% CO_2_), hydrogen abundances as (mol% H_2_)/(mol% CO_2_) and oxygen abundances as (mol% O_2_)/(mol% CO_2_) (hereafter CH_4_/CO_2_, H_2_/CO_2_ and O_2_/CO_2_ respectively). Analyses were only treated as valid if they yielded an order of magnitude more total gas than (that is, at least 20 × ) the blanks. The mass spectrometer has a range of linearity up to 10e−6 amps. The bursts from the meteorites are too low to reach this part of the current range where non-linearity begins, so linearity is assured. Before analysis, the crushing area and the bellows of the crusher were cleaned using potassium hydroxide. The potassium hydroxide was then removed by swabbing with deionized water, followed by air drying. The apparatus is also routinely cleaned with isopropanol. Thereafter, the crushing chamber is baked at about 150–200 °Celsius for 72 h before loading and analysing the samples at room temperature the next day. The crushing area is isolated from the main chamber so that the main chamber can be baked out every evening. This way the samples are not heated overnight and lose gas, but they are exposed to high vacuum to remove adhered gases. Meteorite samples were transferred directly onto hard metal disks for crushing to avoid handling contamination.

### Calibration using blank analysis

A standard NB84, consisting of calcite containing a 25.0% carbon dioxide-water mix, was analysed 52 times. The weighted mean content of carbon dioxide measured was 25.18 mol%, within <1% of the standard composition. This standard is used to calibrate water-gas ratios, but is not essential to measurement of the relative proportions of gases reported in this study. Three blank/reference sample measurements are considered in the measurement of gases. First, a blank is measured from moving parts (bellows in crushing mechanism), without crushing a sample. Second, a procedural blank is measured from crushing a Cenozoic olivine basalt (Beinn Totaig Group, Loch Beag, Island of Skye, Scotland; ref. 53), after heating for 4 h at 750 °C in a vacuum, to cause complete degassing. Third, a blank is measured from crushing synthetic quartz free of visible inclusions (so none larger than 1 μm, if any are present).

The compositions of the quartz blank and basalt blank (mean and standard deviation) are given in Table 1.

The blank from the bellows is negligible compared with the blanks from crushing and is so small that we cannot quantify it. The meteorite yields are much greater than the basalt and quartz blanks, so we have confidence that the yields are predominantly gas from the meteorites rather than an artefact of crushing. The mean basalt blank gives yields 0.03 to 0.10% (mean 0.07%) of the meteorite yields of methane after blank correction (Table 2). The maximum basalt blank (uppermost value of the standard deviation) gives yields 0.68 to 2.30% of the meteorite yields. The procedural blank was deducted from measurements, excepting for carbon dioxide that was measured from the quartz blank, as the heating-degassing process generates new carbon dioxide.

The blanks quantify the maximum possible contribution of trace gas produced by the crushing process, which we attribute to the metal crushing discs. The blank current is about double that of the background current. The s.d. of the mass spectrometer's signal to noise ratio at the mass 15 background is 1.196e−13.

### Detection limits

The detection limit measurements are as described in ref. 50. The formula is the same as used by ref. 54 and slightly modified by later workers^55^,^56^. The s.d. of an individual determination of the background of a sample is given by the formula:


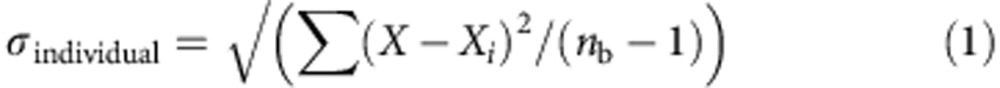


where *X* is the mean, and *X*_*i*_ is one of the values contributing to the mean.

Previous workers^50^,^54^ recognized for a MS transient sampling signal that the measurement error included both elements of the background (*n*_b_ is number of cycles obtained from the background) and the analyte (*n*_a_ is number of cycles used for the measurement of the analyte signal) where the error associated with the net (analyte minus background) signal (*σ*_Rnet_) equals the 1*σ* from the background times the square root of 1/*n*_b_ plus 1/*n*_a_ as shown in the formula below. For *σ*_Rnet_, the s.d. of the mean net signal (gross—background), both *n*_b_ and *n*_a_ are 10 thus improving the *σ*_Rnet_ by 0.45 times the *σ*_*individual*_.


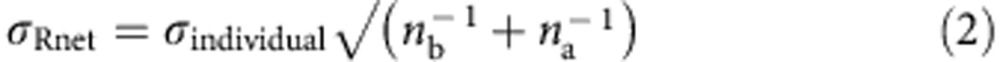


The term *σ*_Rnet_ is the s.d. of the mean net signal and while it is common practice by geochemists to make 2*σ* as the confidence limits or approximately a 95% confidence limit, we adopt a 99% or ∼3*σ* value is used to confirm that the gas burst contains more than zero concentration. As the above s.d. is in terms of signal count rates or other convenient units, it is more useful to convert this value to concentration units by dividing by the sensitivity, *S* (signal per unit concentration):


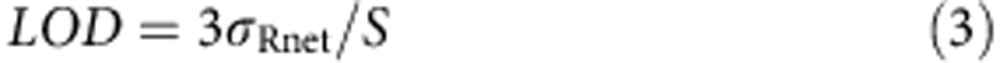


The sensitivity factors, relative to N_2_=1.0, determined at the time of analysis were:


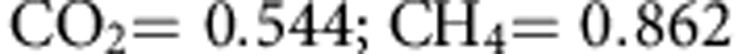


For the mass spectrometer system in its current configuration the 2*σ* detection limit ranges from 0.8 to 1.2 femtomol (10^−15^ mol), depending on noise within the mass spectrometer.

### Introduction to error calculations

Total errors are calculated from six components: blank compositions; uncertainty in gas composition; analytical errors in measuring current; interferences in measuring gas species; error in linear range; and background instrument noise. There is an additional, seventh, component, which only applies to the summary data table (Supplementary Table 2), where mols are reported, based on the conversion of the mass spectrometer current into mols.

### Blank compositions

The s.d. (1 sigma) of blank compositions, as determined above, expressed as current (amps), are:


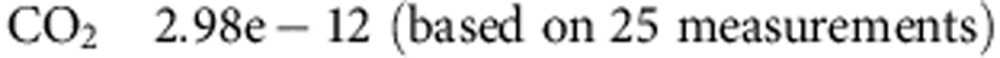



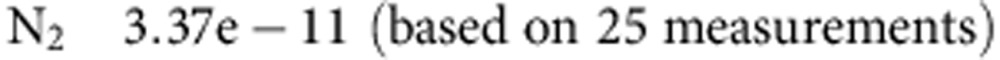



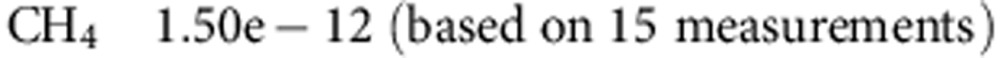


### Uncertainty in gas composition

During calibration, Scott Gas standard gas mixtures are used for the gases H_2_, He, CH_4_ and CO_2_, for which the manufacturer specifies a 2% uncertainty. The gases N_2_, O_2_ and Ar are sourced from the atmosphere for which no uncertainty is assigned. Our measurements of the composition of CH_4_, CO_2_ and N_2_ have a s.d. of 0.061, 0.062 and 0.043% respectively, determined over 400 acquisitions.

### Analytical errors

To quantify the error associated with the incremental crush method, we used atmosphere sealed in seven gas capillary tubes and analysed the N_2_/Ar ratios. The analytical mean ratio was 83.4 and s.d. 0.4; atmosphere is 83.6. The error therefore includes estimation of the area under the peak curve as well as signal noise within the mass spectrometer. Note that, although some analysts using LA-ICP-MS apply a Gaussian formula^57^, gas signals released from fluid inclusions are not Gaussian in shape (also recognized using LA-ICP-MS^55^,^58^,^59^) and our estimation of the area under the curve bears a close resemblance to that of ref. 55. Our 1*σ* precision is 0.5%.

### Interferences in measuring gas species

Three interferences are potentially relevant. First, analysts may consider analysing for CH_4_ at *m*/*e*=16 (which is the major peak); however, this mass corresponds to a minor interference by a fragmented water ion. We used *m/e*=15 instead of 16 for methane as the *m/e*=15 peak (CH_3_+) is 90% of the *m/e*=16 peak and avoids interference corrections from water. The trade-off of 10% increased error at *m/e*=15 versus interference from water makes the *m/e*=15 much more attractive for methane analysis. Second, a very minor interference of 0.032% is detected at the *m/e*=32 (principle mass for oxygen) and is attributable to CO_2_. No adjustment is made for this very low value. The principle interference that occurs is that of CO_2_ fragmentation to produce CO+ and which corresponds to the N_2_ *m/e*=28 channel. Of the total CO_2_ signal, 8.2% is due to CO+ at *m/e*=28. However, typically the CO_2_ is half that of N_2_ for the meteorite samples and therefore the contribution of CO+ to the *m/e*=28 signal is only about 4%. The CO_2_ content at *m/e*=44 is used to remove 4% contribution to the N_2_ value. The relative error added to N_2_ includes a square function and therefore CO_2_ only contributes about 0.16% to the relative N_2_ error.

### Error in linear range

The error across the linear range of the mass spectrometer is estimated from the s.d. for capillary tube measurements of the N_2_/Ar ratio. This covers the noise and the area under the peak curve. The measurements indicate a maximum error of 1%.

### Background instrument noise

The background instrument noise is measured when no sample is being analysed. The signal is measured with the sample exposed to the main chamber and represents all residual gases that comprise the background with the turbo pump operating at maximum speed of 90,000 r.p.m. The mass spectrometer is instructed to begin recording and cycle through the selected masses relating to the gases being analysed. Approximately 30 s later the incremental crush occurs. The background is measured for 10 cycles stopping at 1 s before the gas burst. Both the background signal mean and s.d. are recorded.

### Conversion of current to moles

Conversion from mass spectrometer current (amps) to gas concentration (mols) is achieved by analysing the contents of a Millipore one microliter volume capillary tube containing atmosphere at standard temperature and pressure, sealed using a high-vacuum epoxy glue. The tube is crushed in the same manner as any other sample. The number of moles of nitrogen (obtained using PV=nRT) is equated to the measured current. The equivalence determined at the time of measurement of the meteorites was 1,054 amps per mol, calculated by conventional matrix methods^51^,^52^. The uncertainty in measurement is no more than 5%, based on the s.d. of measurements of the ratio of current to moles calculated from the area under the peak curve. In addition, the manufacturer reports a maximum 1% uncertainty in the volume of the capillary tube. Adding these two errors give 5.04% (square root of added squares).

### Propagation of errors

The uncertainty matrix includes multiple components as described above. There will be different contributions of uncertainties for each gas. The formula to incorporate the additive uncertainties is:


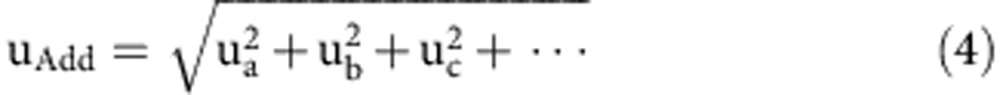


The components used in the additive error calculation are given in Table 3.

An example of the errors is given in Table 4. This is for a crush of the Zagami meteorite and is the smallest burst of gas measured in the programme.

## Additional information

**How to cite this article:** Blamey, N.J.F. *et al.* Evidence for methane in Martian meteorites. *Nat. Commun.* 6:7399 doi: 10.1038/ncomms8399 (2015).

## Supplementary Material

Supplementary Information Supplementary Tables 1-2, Supplementary Notes 1-2 and Supplementary References

## Figures and Tables

**Table 1:** Composition of quartz and basalt blanks.

**Gas**	**Qtz blank mean (mols)**	**Qtz blank s.d. (mols)**	**Basalt blank mean (mols)**	**Basalt blank s.d. (mols)**
H_2_	5.1E−15	1.31E−14	3.01E−15	4.09E−15
He	Below detection	0	2.12E−16	2.50E−16
CH_4_	4.14E−14	2.08E−14	6.62E−16	1.42E−15
N_2_	2.84E−15	3.2E−14	1.14E−12	6.81E−13
O_2_	1.1E−15	4.7E−16	0	0
Ar	1E−16	1.6E−16	3.01E−16	5.37E−16
CO_2_	4.5E−15	2.83E−15	5.50E−11	4.27E−11

**Table 2:** Blank methane value as percentages of meteorite methane values.

**Sample**	**Blank % of meteorite**	**Blank+s.d. % of meteorite**
NWA5790	0.08	1.76
Zagami	0.04	0.90
LA002	0.10	2.30
Nakhla	0.05	1.04
Y000749	0.03	0.68
MIL03346	0.10	2.26

**Table 3:** Components used in additive error calculation.

	**CO**_**2**_	**N**_**2**_	**CH**_**4**_
Blank (current (amps), 1 sigma s.d.)	2.98e−12	3.37e−11	1.50e−12
Uncertainty in gas composition from standard	2%	0	2%
Interference	0	4% of CO_2_	0
Analytical error	0.5%	0.5%	0.5%
Error in linear range	1.0%	1.0%	1.0%
Background instrument noise (1-sigma)	Specific to analysis	Specific to analysis	Specific to analysis
Conversion current to mols	5.04%	5.04%	5.04%

**Table 4:** Errors calculated for smallest crush of the Zagami meteorite.

**Source of error/uncertainty**	**CO**_**2**_	**N**_**2**_	**CH**_**4**_
Background instrument noise (1-sigma)	5.20e−13	1.47e−12	1.05e−13
Blank (1-sigma)	2.98e−12	3.37e−11	1.50e−12
Uncertainty in gas composition from standard	4.73e−13	0	1.19e−11
Interference	0	2.92e−13	0
Analytical error (0.5%)	1.18e−13	4.75e−13	2.98e−12
Error in linear range (1%)	2.36e−13	9.50e−13	5.96e−12
Additive error before converting to mols	3.07e−12	3.37e−11	1.37e−11
Conversion current to mols for this crush	3.13e−15 mols	3.24e−14 mols	3.14e−14 mols
