# Supplementary material for: Evidence for methane in Martian meteorites
Source: Nat Commun. 2015 Jun 16;6:7399. doi: 10.1038/ncomms8399 (PMC4521231; doi:10.1038/ncomms8399)
Supplement: Supplementary Information — Supplementary Tables 1-2, Supplementary Notes 1-2 and Supplementary References [file ncomms8399-s1.pdf]

Supplementary Table 1. Summary of meteorites sampled

The six samples analysed were as follows:

Meteorites used for volatile analysis

Meteorite	Sub-group	Crystallization age (Ma)	Ejection age (Ma)	Terrestrial age (Ma)	Pyroxene/olivine*	Fe contents
Nakhla	nakhlite	1380±70	10.8±0.8	Fall	74-85% pyr, 5-16% ol	Fs_{20-40}, Fa_{65-75}
MIL 03346	nakhlite	1360±30	9.5±1.0	unknown	65-80% pyr, 3% ol	Fs_{20-55}, Fa_{57-95}
NWA 5790	nakhlite	1380±100	~9.6	unknown	51% pyr, 9% ol	Fs_{24-47}, Fa_{65-88}
Y 000749	nakhlite	1367±7	12.1±0.8	<0.04	77-85% pyr, 10-12% ol	Fs_{20-70}, Fa_{62-78}
Zagami	shergottite	177±3	3.0±0.3	Fall	70-80% pyr, trace ol	Fs_{10-45}, Fa_{90-92}
LA 002	shergottite	170±8	3.0±0.3	unknown	40% pyr, trace ol	Fs_{30-70}, Fa_{95}

Pyr % pyroxene, ol % olivine, Fs % Ferrosilite (iron pyroxene), Fa % Fayalite (iron olivine).*Excludes additional pyroxene and olivine in mesostasis

Sources:

Mars Meteorite Compendium[1]. Data for Y 000749 partially from paired Yamato meteorite Y 000593.

Ages from Supplementary References 2-5.

Supplementary Table 2. Summary data table (blank mean and 1-std deviation subtracted)

The summary data table reports the total gas yield from all incremental crushes per sample applying the weighted mean and weighted standard deviation (analysis of multiple incremental crushes; contact the author directly for the analyses of individual crushes). The mean is weighted by the signal size in mol% divided by the mass spec amps/mol ratio (which is 1054 as noted above) to provide the mols of gas. The standard deviation reported in the table incorporates (i) a largest contribution from the weighted standard deviation of analyses for all incremental crushes, (ii) the 5.04% error in conversion from current to moles, and (iii) a third, very minor contribution to the overall standard deviation from the error propagation used for individual incremental crushes, incorporating the six components itemised above (blank composition, uncertainty in gas composition, analytical errors, interferences in measuring gas species, error in linear range, background instrument noise) which contributes less than 1% to the overall standard deviation. These three components are combined by a second application of the additive formula given in the Methods section.

Name	Type	Mass (g)	Total amount of gas released in moles (standard deviation)							Weighted mean ratios			
			H_2	He	CH_4	CO_2	O_2	Ar	N_2	**CH_4/CO_2**	**H_2/CO_2**	**O_2/CO_2**	**Ar/CO_2**
**LA002**	Shergottite	0.155	6.16×10^{-13}<br>(4.58×10^{-13})	1.38×10^{-15}<br>(1.57×10^{-15})	3.46×10^{-12}<br>(2.26×10^{-12})	1.14×10^{-12}<br>(9.40×10^{-13})	9.21×10^{-14}<br>(9.31×10^{-14})	1.68×10^{-13}<br>(8.01×10^{-14})	3.30×10^{-11}<br>(4.97×10^{-12})	3.04<br>+18.3<br>−2.34	0.54<br>+3.52<br>−0.450	0.0808<br>+0.624<br>−0.0797	0.147<br>+0.783<br>−0.100
**Nakhla**	Nakhlite	0.124	4.33×10^{-13}<br>(2.07×10^{-13})	3.60×10^{-17}<br>(6.51×10^{-17})	4.36×10^{-12}<br>(1.21×10^{-12})	8.32×10^{-13}<br>(5.05×10^{-13})	4.04×10^{-14}<br>(1.41×10^{-14})	1.73×10^{-14}<br>(1.62×10^{-14})	1.36×10^{-12}<br>(7.91×10^{-13})	5.24<br>+8.95<br>−2.57	0.52<br>+0.0946<br>−0.0271	0.048<br>+1.26<br>−0.362	0.0208<br>+0.0675<br>−0.0193
**NWA5790**	Nakhlite	0.155	3.81×10^{-13}<br>(3.68×10^{-13})	2.49×10^{-15}<br>(3.50×10^{-16})	1.74×10^{-12}<br>(4.50×10^{-13})	6.04×10^{-13}<br>(1.85×10^{-13})	3.06×10^{-14}<br>(2.28×10^{-13})	1.78×10^{-14}<br>(4.28×10^{-15})	1.63×10^{-12}<br>(2.72×10^{-13})	2.88<br>+1.75<br>−1.04	0.63<br>+0.275<br>−0.163	0.0507<br>+0.0227<br>−0.0135	0.0295<br>+0.0177<br>−0.0105
**Mil 03346**	Nakhlite	0.126	1.09×10^{-12}<br>(4.04×10^{-13})	2.89×10^{-16}<br>(2.82×10^{-16})	2.75×10^{-12}<br>(4.88×10^{-13})	9.46×10^{-13}<br>(6.29×10^{-13})	2.65×10^{-14}<br>(3.24×10^{-14})	6.77×10^{-15}<br>(4.84×10^{-15})	2.26×10^{-13}<br>(3.38×10^{-13})	2.91<br>+5.43<br>−1.31	1.15<br>+2.89<br>−0.695	0.0280<br>+0.130<br>−0.0314	0.00716<br>+0.0242<br>−0.00583
**Zagami**	Shergottite	0.147	2.18×10^{-12}<br>(2.24×10^{-12})	1.42×10^{-15}<br>(1.29×10^{-15})	1.02×10^{-11}<br>(2.94×10^{-12})	4.45×10^{-13}<br>(2.50×10^{-13})	4.93×10^{-14}<br>(2.21×10^{-14})	1.82×10^{-14}<br>(1.11×10^{-14})	2.88×10^{-12}<br>(2.97×10^{-12})	22.9<br>+0.252	4.90<br>+13.9	0.111<br>+0.196	0.0409<br>+0.0851

										−0.184	−4.69	−0.00662	−0.0287
**Y000749***	Nakhlite	~ 0.1	1.84×10^{-12}	Below detection	4.36×10^{-12}	3.35×10^{-13}	Below detection	5.52×10^{-15}	Below detection	13.0	5.48	N/A	0.016
**Murchison**	Carbonaceous chondrite	~ 0.1	3.39×10^{-12}<br>(2.21×10^{-12})	1.63×10^{-15}<br>(2.85×10^{-15})	7.46×10^{-12}<br>(5.44×10^{-12})	4.98×10^{-11}<br>(1.48×10^{-11})	9.23×10^{-13}<br>(4.29×10^{-13})	3.80×10^{-13}<br>(7.74×10^{-13})	1.18×10^{-10}<br>(3.41×10^{-11})	0.150<br>+0.185<br>−0.111	0.0681<br>+0.0773<br>−0.0465	0.0185<br>+0.01644<br>−0.00990	0.00763<br>+0.00227<br>−0.0137
**Tagish Lake**	Carbonaceous chondrite	~ 0.1	1.53×10^{-13}<br>(8.96×10^{-14})	Below Detection	1.26×10^{-12}<br>(3.62×10^{-13})	1.20×10^{-11}<br>(1.19×10^{-12})	1.11×10^{-13}<br>(3.07×10^{-14})	Below detection	2.13×10^{-12}<br>(3.62×10^{-13})	0.105<br>+0.0307<br>−0.0283	0.0128<br>+0.00787<br>−0.00726	0.00925<br>+0.00258<br>−0.00238	N/A

* Two bursts only

Supplementary Notes 1. Sample provenance

The provenance of the samples is as follows:

Zagami

Loaned by the Institute of Meteoritics at the University of New Mexico (sample UNM 992).

Zagami is a Shergottite which is roughly basaltic in composition and shows signs of heavy shock. It is composed of approximately 75% augite/pigeonite, 17% maskelynite, 3% titanomagnetite, 2% quenched mesostasis, 1% fayalite (~Fa_{90}), 1% pyrrhotite and minor amounts of phosphates. It comprises closely packed pyroxene crystals intergrown with maskelynite and quenched mesostasis material along with minor phases, and displays a subcumulate texture. The fayalite in Zagami is present as late-stage intergrowth among other phases.

Nakhla

First Nakhla sample Loaned by Dr. Caroline Smith, Meteorite Curator, British Natural History Museum (Sample BM1913_25)
Donated by Dr. David Deamer at the University of California Santa Cruz.

Nakhla is a clinopyroxenite and displays a cumulate texture. It comprises pyroxene and olivine crystals intergrown with quenched mesostasis material and minor phases. It shows signs of having undergone minimal shock. It is composed of approximately 70% augite, 15% fayalite (~$Fa_{65\text{-}75}$), 10% quenched mesostasis material, 3% titanomagnetite, 1% pyrrhotite, 1% Fe-Mg alteration assemblages (iddingsite), anhydrite, gypsum, halite and minor amounts of carbonates. The fayalite in Nakhla is present as primary crystals ~1 mm in diameter.

Los Angeles 002

Donated by Robert Verish of the Meteorite Recovery Laboratory in California.

Los Angeles 002 is a Shergottite which is roughly basaltic in composition. It comprises pyroxene crystals intergrown with large maskelynite zones and quenched mesostasis material; minor phases are intergrown with the dominant phases. It is composed of approximately 35% augite/pigeonite, 40% maskelynite, 10% pyroxferroite (iron-rich breakdown products of metastable primary pyroxene phases), 3% quenched mesostasis material, 3% titanomagnetite, 3% fayalite (~Fa_{95}), and minor amounts of silica and phosphates. The fayalite in Los Angeles 002 is present as intergrown material among other phases.

North West Africa 5790

Obtained from Darryl Pitt, Curator of the Macovich Collection based in New York

North West Africa (NWA) 5790 is one of the nakhlite meteorites and is a cumulate rock. Like other nakhlites it appears to have undergone minimal shock. It is composed of 51% augite, 9% fayalite (Fa_{65-80}), 40% quenched mesostasis and <1% titanomagnetite with minor iddingsite. The outer surface of NWA 5790 is observed to be covered with caliche[6]. The fayalite crystals found within the outer edges of the sample are coated with terrestrial alteration products (calcite, phyllosilicate). However, iddingisite-like clays interpreted to be pre-terrestrial are observed underlying terrestrial contamination but in some cases are partially weathered.

Yamato 000749

Loaned by Professor Hideyasu Kojima, Meteorite Curator, National Institute of Polar Research, Japan (sample Yamato 000749 [59])

Yamato (Y) 000749 is one of the nakhlites which are cumulate rocks. It is paired with Y 000593 and Y 000802. Like other nakhlites it appears to have undergone minimal shock. It is composed of 71% augite 18% fayalite (Fa_{60-83}), 10% mesostatsis and minor amounts of titanomagnetite and alteration assemblages[7]. The fayalite crystals are <0.5 mm in diameter. Ferriolivine laihunite (known to form terrestrially between 400-800°C) is observed along the edges of fayalite cracks and is thought to predate the iddingsite, thereby confirming its martian origin[8]. Jarosite is also observed within Yamato 000749; however, its true origins (terrestrial or martian) are unclear.

Miller Range 03346

Loaned by The Meteorite Working Group, NASA Johnson Space Center (sample MIL 03346, 118 [205])

Miller Range (MIL) 03346 is one of the nakhlites which are cumulate rocks. It is paired with MIL 090030, 090032 and 090136. Like other nakhlites it appears to have undergone minimal shock. It is composed of 78% augite, 3% fayalite (Fa_{57-86}), 17% mesostasis, and 1% titanomagnetite with minor amounts of silica, sulphate, sulphides and olivine alteration products (iddingsite and laihunite). MIL 03346 is thought to have experienced minimal shock (similar to all nakhlites). The fayalite crystals are <1.5 mm in diameter. Jarosite and gypsum are observed to be abundant within the exposed outer edges of the MIL 03346 samples. Based on these observations it is thought that majority of the sulphates within MIL are terrestrially derived[9].

Supplementary Notes 2. Sample level of thermal alteration

The degree of shock of Nakhla, Governador Valadares, Lafayette and Y-000593 suggests that their temperatures would have been elevated by 20±10 K at most during impact-ejection from Mars[10]. Using $^{40}Ar/^{39}Ar$ thermochronometry, Miller Range 03346 has not been significantly heated since crystallization[11], whereas Nakhla experienced only a localised and brief heating event at 913 ± 9 Ma, a long time before its transfer to Earth. Other evidence for a lack of heating is retention of martian water in the iddingsite of Nakhla and Lafayette[12,13]. The methane in the meteorite samples was, therefore, not generated by heating.

Supplementary References

1. Mars Meteorite Compendium. http://curator.jsc.nasa.gov/antmet/mmc/index.cfm

2. Park, J., Garrison, D.H. & Bogard, D.D. 39Ar-40Ar ages of martian nakhlites. *Geochim. Cosmochim. Acta* **73**, 2177-2189 (2009).

3. Murty S. V. S., Mahajan R. R., Goswami J. N. & Sinha N. Noble gases and nuclear tracks in the nakhlite MIL 03346. *Lunar Planet. Sci. Conf.* **XXXVI**, 1280 (2005).

4. Huber L., Irving, A.J., Maden, C. & Wieler, R. Noble gas cosmic ray exposure ages of four unusual Martian meteorites: Shergottites NWA 4797, NWA 5990, NWA 6342 and Nakhlite NWA 5790. *Lunar Planet. Sci. Conf.* **XLIII**, 1408 (2012).

5. Okazaki, R., Nagao, K., Imae, N. & Kojima, H. Noble gas signatures of Antarctic nakhlites, Yamato (Y) 000593, Y000749 and Y000802. *Antar. Meteor. Res.***16**, 58-79 (2003).

6. Shih, C.-Y., Nyquist, L.E. & Jambon, A. Sm-Nd isotopic studies of two nakhlites, NWA 5790 and Nakhla. *Lunar Planet. Sci. Conf.* LXI,**1367** (2010).

7. Imae, N., Ikeda, Y., Shinoda, K., Kojima, H. & Naoyoshi, I. Yamoto nakhlites: Petrography and mineralogy. *Antar. Meteor. Res.* **16**, 13-33 (2003).

8. Noguchi, T. *et al.* Laihunite and jarosite in the Yamato 00 nakhlites: alteration products on Mars? *J. Geophys. Res.* **114**, E10004, doi:10.1029/2009JE003364 (2009).

9. Hallis, L.J. & Taylor, G.J. Comparisons of the four Miller Range nakhlites, MIL 03346, 090030, 090032 and 090136: Textural and compositional observations of primary and secondary mineral assemblages. *Meteor. Planet. Sci.* **46**, 1787-1803 (2011).

10. Fritz J., Artemieva N. & Greshake A. Ejection of Martian meteorites. *Meteor. Planet. Sci.* **40**,1393–1411 (2005).

11. Cassata W. S., Shuster D. L., Renne P. R. & Weiss B. P. Evidence for shock heating and constraints on Martian surface temperatures revealed by 40Ar/39Ar thermochronometry of Martian meteorites. *Geochim. Cosmochim. Acta* **74**, 6900–6920 (2010).

12. Leshin L.A., Epstein S. & Stolper E.M. Hydrogen isotope geochemistry of SNC meteorites. *Geochim. Cosmochim. Acta* **60**, 2635-2650 (1996).

13. Hallis, L.J. *et al.* Hydrogen isotope analyses of alteration phases in the nakhlite martian meteorites. *Geochim. Cosmochim. Acta* **97**, 105–119 (2012).
